# Editorial: Biosafety of Genetically Modified Organisms 3. A Collection of Publications from the 15th International Society for Biosafety Research Symposium

**DOI:** 10.3389/fbioe.2021.745937

**Published:** 2021-08-26

**Authors:** Karen Hokanson, Alan Raybould, Andrew Roberts, Jörg Romeis, Joe Smith

**Affiliations:** ^1^ Department of Horticultural Science, University of Minnesota Twin Cities, Minneapolis, MN, United States; ^2^ Innogen Institute, School of Social and Political Science, The University of Edinburgh, Edinburgh, United Kingdom; ^3^ Global Academy of Agriculture and Food Security, Easter Bush Campus, The University of Edinburgh, Edinburgh, United Kingdom; ^4^ Agriculture and Food Systems Institute, Washington, CA, United States; ^5^ Agroscope (Switzerland), Zürich, Switzerland; ^6^ Independent researcher, Canberra, ACT, Australia

**Keywords:** biosafety, regulation, risk assessment, sustainable biotechnology, policy innovation

**FIGURE 1:**
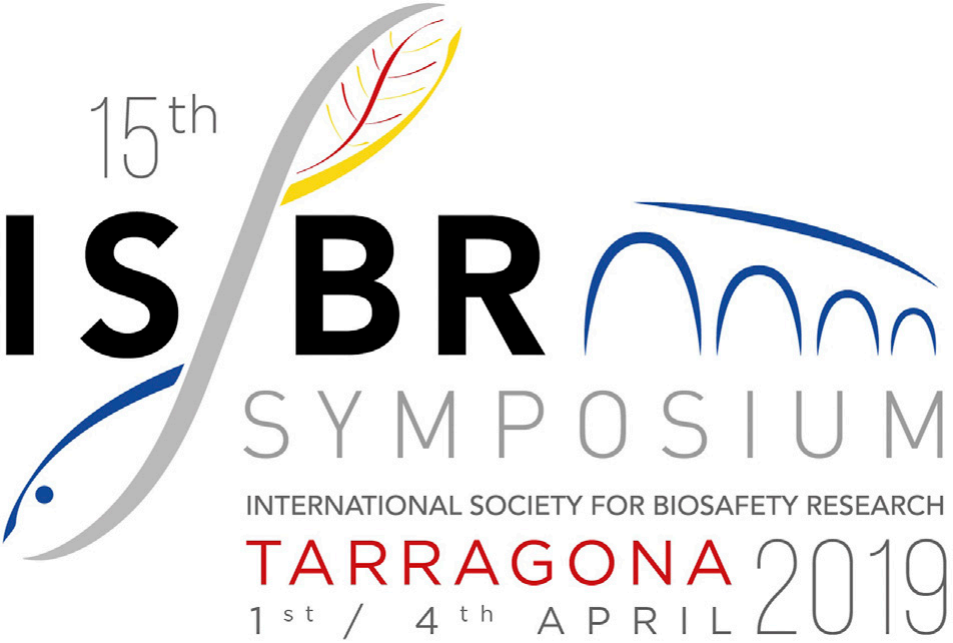
The 15th ISBR Symposium was held in April 2019 in Tarragona, Spain.

The ISBR Symposium [previously known as the International Symposium on Biosafety of Genetically Modified Organisms (ISBGMO)] is an international meeting organized by the International Society for Biosafety Research (ISBR), a society whose membership is composed of individuals with expertise and interest in regulations, risk assessments, and research associated with the sustainable use of biotechnology (http://www.isbr.info/). These symposia have been offered biennially since 1990, at various locations throughout the world, as a unique opportunity for public and private sector research scientists, regulators, technology developers, nongovernment organizations and others to share their experience and expertise and to discuss biosafety related to the application of biotechnology. As with past symposia, ISBR hosted a research topic titled “Biosafety of Genetically Modified Organisms 3” in Frontiers in Bioengineering and Biotechnology: section Biosafety and Biosecurity, open to the presenters at the most recent 15th ISBR Symposium held in April of 2019 in Tarragona, Spain (Figure 1).

The goals of the ISBR Symposium are 1) to share biosafety research and application and chart new research directions, 2) to foster productive dialogue and multidisciplinary approaches, and 3) to embrace perspectives from all parts of the globe. The emphasis by ISBR for these symposia has evolved over the years. Early symposia were focused mainly on presentations of the results of research related to risks of biotechnology and environmental risk assessment. More recent meetings have increasingly included presentations, workshops, and forward-thinking discussions on the regulation of biotechnology, data requirements, and the relevance of risk assessment research for decision-making, policy development and encouraging innovation. This shifting emphasis highlights the distinctiveness of this symposium compared to other more academic scientific meetings that may feature biosafety among their sessions. ISBR also has intentionally broadened the scope of the symposia to include new and emerging applications of biotechnology with implications for regulatory research and policy that are different than the “traditional” genetically modified organisms of the past.

The 15th ISBR Symposium, with 274 attendees participating from 42 countries, had as its theme “New Horizons in Biotechnology: Risk Analysis for a Sustainable Future”. Around this central theme, the program included a series of presentations in four topical plenary sessions: 1) Communications and Engagement with Policy and Public Audiences; 2) Environmental Risk Assessment and Regulation of Gene Edited Products; 3) Food Safety Assessment of Novel Molecules–What Does the Future Hold; and 4) Challenges in the Development and Adoption of Novel Biotechnologies. In addition to these plenary sessions, the symposium included over 20 organized sessions and workshops offered in parallel on a range of topics, numerous Pecha Kucha and traditional poster presentations, and accommodated some smaller satellite conferences in its margins. Out of the diverse presentations, the society has assembled a collection of 17 peer-reviewed publications representative of the different kinds of thought-provoking topics presented and discussed at the ISBR symposium.

The research topic from the 15th ISBR Symposium includes four timely and compelling “policy and practice reviews,” perhaps most representative of the novelty of the ISBR Symposium. Two of these articles deal specifically with the most current new breeding techniques broadly described as “gene-editing” that have garnered a great deal of attention and discussion among the biotechnology research and regulatory communities. In fact, one of the plenary session topics at the symposium was devoted to environmental risk assessment and regulation of gene-edited products. One of the reviews is based on a presentation in that plenary session and presents an important discussion of the regulatory experience in Argentina with gene-edited products compared to “traditional” GMOs (genetically modified organisms), and the potential for changes in regulatory practices to encourage innovation (Whelan et al.). The second review describes Japan’s progress in developing and implementing a regulatory approach for genome-edited organisms within the existing biosafety framework (Tsuda et al.). Governments worldwide are similarly working to define their approach to regulating gene-edited organisms, recognizing the great potential for applications of this technology in human health and agriculture.

Another important policy and practice review discusses regulation of GMOs being developed for invasive species control, specifically using gene drive applications (Mitchell and Bartsch). Gene drives are another hot regulatory topic because of the increased potential for transboundary movement and replacement of target populations associated with this technology. This article identifies information gaps and considers scenarios for safely releasing gene drive organisms into the environment. The fourth policy and practice review takes a close look at biosafety and biosecurity of GMOs in containment and provides a global overview of how regulatory frameworks have evolved to manage these (Beeckman and Rüdelsheim). This article includes a very useful discussion of different ways biosafety and biosecurity can be defined and the scope of biosafety as it overlaps with biosecurity.

The research topic also includes three progressive general “review” articles. Teem et al. describe different approaches for genetic biocontrol, including gene drives, and the regulatory considerations of each to minimize potential harm to the environment. Another review discusses the deliberations taking place on “synthetic biology” under the Convention on Biological Diversity (CBD), a multilateral treaty that has significant implications for regulation of biotechnology (Keiper and Atanassova). This review describes synthetic biology as “part of the continuum of modern biotechnological development”; as such, the adequacy of existing regulatory mechanisms for “living modified organisms” to also regulate the “new” biotechnologies encompassed by “synthetic biology” has become central to the CBD deliberations. A third article reviews ongoing discussions about the appropriate tests and use of endpoints needed to inform non-target arthropod assessment of crop plants with pesticidal properties, especially for new technologies that have a different mode of action than the more familiar Bt Cry proteins such as traits based on RNA interference (Roberts et al.).

The 15th ISBR Symposium research topic includes four ‘original research’ articles. Two of these address some of the most common environmental and food safety concerns associated with genetically engineered crops. Xu et al. present the results of a study to understand the potential risk to nontarget organisms due to changes in herbivore-induced plant volatiles of an insect resistant Bt maize compared to non-Bt maize. This study concluded that the changes in plant volatiles do not affect the behavior of *Trichogramma* egg parasitoids, considered beneficial in controlling lepidopteran pests of maize. Bressan et al. studied the potential occurrence of gene flow from sugarcane cultivars to wild relatives and the nutritional composition of sugarcane cultivars in Brazil, to use as baseline studies for risk assessment of genetically engineered sugarcane.

Another article, describing original research that has received marked attention, reports on the first limited field release of a genetically engineered, “self-limiting” agricultural pest insect, the diamondback moth which is a serious global pest of crucifers, and the series of studies that were conducted to evaluate its potential as a biologically-based approach to crop pest management (Shelton et al.). In addition, an article (by the same first author) presents the results of research demonstrating the impact in the market value chain in Bangladesh of a genetically engineered insect resistant brinjal (eggplant), one of the first genetically engineered food crops approved for cultivation in a developing country (Shelton et al.). This article was complemented by a “brief research report” considering the biosafety management measures as well as socio-economic impacts and challenges of this same insect resistant brinjal in Bangladesh (Haque and Saha).

The remaining articles in the 15th ISBR Symposium research topic are five “perspective” pieces. Perspectives are welcomed additions to the research topic, as these short articles offer an opportunity for authors to capture their thoughts and experiences on specific topics as presented at the symposium. Four of these perspectives come from Latin America and each of these shares lessons that should be applicable to regulatory systems across the globe. One of these discusses how different countries in the Americas have applied the concept of familiarity in risk assessments of transgenic crops and effectively demonstrates how this concept has become a key element of the risk assessment process (Capalbo et al.). Another paper describes the establishment in the regulatory system of Paraguay of a simplified procedure for evaluating the safety of GM crops that allows the use of risk assessments and decision documents already issued in another country for the same GM event (Candia et al.). A similar idea, the transportability of conclusions from confined field trials from Brazil to Argentina, is discussed in a perspective that uses the virus resistant GM bean developed in Brazil as a case study (Vesprini et al.). Another perspective is shared in an article from Argentina that discusses the challenge for locally developed GM crops to reach the market compared to those coming from private industry, and the need for a regulatory affairs platform for the public research system (Lewi and Vicién). One more perspective describes the experience of developing an effective insect resistance management (IRM) strategy for Bt maize following the discovery of resistance development in the target insect pest in South Africa, with implications for developing more effective IRM strategies for other insect resistant maize in Africa (Bouwer).

ISBR gratefully acknowledges the contribution from all the authors to this research topic. The society has identified an important niche to fill in the scientific community, and the diversity of topics and article types published as part of this research topic exemplify the goals and impact of the ISBR Symposium. The society intends to continue to bring together this unique group to share perspectives, learn from experiences and plan for sound scientific global approaches to biosafety in the future. The 16th ISBR Symposium will take place in April 2022 in St. Louis, Missouri United States.

